# Association between flavonoids intake and dental caries in children and adolescents: a cross-sectional study from the NHANES database

**DOI:** 10.1186/s12903-024-04567-1

**Published:** 2024-07-26

**Authors:** Jianing Fan, Siqi Liu, Qian Zhang, Li Qiao, Qingsong Chu

**Affiliations:** Department of Stomatology, Cangzhou People’s Hospital, Intersection of Huanghe West Road and Qiushi South Road, Cangzhou, 061000 China

**Keywords:** Flavonoids, Dental caries, Children, Adolescents, NHANES database

## Abstract

**Background:**

Worldwide, dental caries is a bacterial biofilm-mediated condition with a high morbidity in children and adolescents. Flavonoids are a class of active natural products with antibacterial and anti-inflammatory effect. In vivo and in vitro studies have shown that they can promote tooth mineralization and reduce inflammation. However, the association of flavonoids intake and dental caries in children and adolescents remain unclear.

**Aim:**

This study was to evaluated the association of flavonoid and its subclass intake and dental caries in children and adolescents.

**Methods:**

Data of participants aged 2-17 years were extracted from the National Health and Nutrition Examination Survey (NHANES) database (2017-2018). Dental caries was measured via the decayed or filled surfaces in primary teeth or permanent teeth (dfs/DFS) index. The weighted univariable and multivariable logistic regression models were utilized to explore the association of flavonoids intake with dental caries in children and adolescents, with odds ratios (ORs) with 95% confidence intervals (CIs). Subgroups analyses based on age, and overweight/obesity were further assessed the association. Subgroup analysis were further performed to explore whether the association between subclasses of anthocyanidins and catechins with dental caries was robust stratified by age and individual with overweight/obesity.

**Results:**

Among totally 1,818 children and adolescents, 786 (43.2%) had dental caries. High intake of anthocyanidins (OR=0.69, 95%CI: 0.52-0.92) and catechins (OR=0.64, 95%CI: 0.44-0.92) were associated with lower odds of dental caries. Similar results were discovered in individuals aged ≥6 years (anthocyanidins, OR=0.62, 95%CI: 0.43-0.90; catechins, OR=0.62, 95%CI: 0.40-0.96), and without overweight/obesity (anthocyanidins, OR=0.58, 95%CI: 0.37-0.90; catechins, OR=0.51, 95%CI: 0.31-0.84). Further investigation found that high intake of cyanidin, petunidin, malvidin, peonidin, (+)-Catechin, (-)-Epigallocatechin, and (-)-epicatechin were associated with lower odds of dental caries in children and adolescents.

**Conclusion:**

High intake of anthocyanidins and catechins were associated with lower odds of dental caries in children and adolescents and are a promising intervention to be further explored in children and adolescents.

**Supplementary Information:**

The online version contains supplementary material available at 10.1186/s12903-024-04567-1.

## Background

Dental caries refers to the loss of enamel and dentin mineral caused by bacterial biofilms, thin bacterial membranes attached to the body surface [1, 2], which is estimated to affect 60%-90% children and adolescents worldwide [3, 4]. Dental caries may lead the softening of hard tooth tissue and the formation of cavities, as well as conditions and symptoms such as pain, infection, abscesses, and sepsis [3], which can produce serious health sequelae and have a negative impact on quality of life [5–8]. There are many influencing factors for an individual to develop dental caries, of which poor dietary habits are one of the main drivers [5, 9–12]. The adjustment of daily diet may be a potentially beneficial measure in preventing dental caries in children and adolescents.

Phytochemicals are dietary anti-oxidants and are an important part of the human diet, including polyphenols, terpenoids, organosulfurs, and phytosterols [13]. Flavonoids, polyphenolic compounds, have gained attention regarding their potential importance for health [14–16]. Evidence showed that flavonoids from healthy diets have multiple biological activities including antioxidant, antibacterial and anti-inflammatory [13, 17–21]. Recent studies have reported new mechanisms underlying the protective effects of flavonoids and their subclasses on dental caries [20, 22–24]. Anthocyanidins and catechins promote tooth remineralization mainly by inhibiting the growth and biofilm formation of streptococcus mutans [20, 23, 25]. Two adult studies on the relationship between flavonoids subclasses and dental caries showed that consumption of green tea with high concentrations of catechins was associated with lower odds of tooth loss [26, 27]. However, current studies on flavonoids and dental caries have focused on adults as well as in vivo and in vitro experiments, and whether there is a benefit of flavonoid intake on dental caries in children and adolescents remains unclear.

This study was to investigate the association of total flavonoids and its subclasses intakes with dental caries in children and adolescents, and to provide reference for the dietary prevention and control of dental caries in children and adolescents.

## Methods

### Study design and participants

Data of participants aged 2-17 years in this cross-sectional study were extracted from the National Health and Nutrition Examination Survey (NHANES) database (2017-2018). The NHANES is a program of studies conducted by the National Center of Health Statistics (NCHS) and the Centers for Disease Control and Prevention (CDC) to assess the health and nutritional status of the civilian and non-institutionalized populations in the United States [28]. Data collection was carried out through interviews, including demographic, dietary, socioeconomic, health-related questions, dental, medical, and physiological measurements, and laboratory tests [29]. NHANES is a publicly available dataset and was approved by the NCHS Ethics Review Board. The requirement of ethical approval for this was waived by the Institutional Review Board of Cangzhou People’s Hospital, because the data was accessed from NHANES (a publicly available database). The need for written informed consent was waived by the Institutional Review Board of Cangzhou People’s Hospital due to retrospective nature of the study. All methods were performed in accordance with the relevant guidelines and regulations.

The inclusion criteria were: (1) 2-17 years old. The exclusion criteria were: (1) missing information of caries measurement; (2) missing complete information of flavonoids intake.

### Assessment of total flavonoids and its subtypes intake

Data of total flavonoids and their subclasses intakes were extracted from the United States Department of Food and Nutrient Database for Dietary Studies (FNDDS) linked to the NHANES database. The flavonoid intake information of subjects aged 2-6 years old were responded by their guardian; subjects aged 7-11 years old were accompanied by their guardian to assist in responding, while subjects aged 12-17 years old were responded by themselves. The mean levels of the compounds were calculated by two 24-h interviews. Totally 29 flavonoids in 6 flavonoid subclasses for all food codes were provided in the United States Department of Agriculture database, which linked the NHANES database (2017–2018) [30]. These data could be utilized to estimate flavonoids consumption in the U.S. population. In present study, the total flavonoids intake was classified as tertiles to explore the association between flavonoid intake and dental caries among childre and adolescents. Then, anthocyanidins and catechines were subdivided into 12 kinds to further explore their association with dental caries. Because more children and adolescents consumed 0 mg of anthocyanin and catechin, the 0 mg intake was divided into one group and the remaining were divided into two groups according to median intake.

### Potential covariates

The potential covariates were extracted as follows: age (<6 or ≥6 years old), gender (female or male), race (Non-Hispanic White, Non-Hispanic Black, or others), poverty income ratio (PIR) (<1.0, or ≥1.0), household reference person education level (<high school, or ≥high school), body mass index (BMI, kg/m^2^), birth weight (<5.5, 5.5-8.9, or ≥9 pounds) [5], smoking during pregnancy, serum cotinine (≤0.05, >0.05 ng/mL, or unknown). BMI at or above the 85^th^ and below the 95^th^ sex-specific percentile of the BMI-for-age growth chart was defined as overweight according to the CDC. Obesity was defined as BMI at or above the sex-specific 95^th^ percentile of the CDC BMI-for-age growth chart. Underweight was defined as having BMI at or above the 5^th^ percentile and normal weight was defined as having a BMI at or above the 5^th^ percentile and below the 85^th^ percentile, for age and gender [31]. Serum cotinine was a reliable nicotine biomarker to objectively measure individuals exposed to tobacco smoke and 0.05 ng/mL was used for cutoff value. Amount of toothpaste use was divided into two groups: <half load and ≥half load according to “How much toothpaste do you use?”. Frequency of tooth brushing was divided into two groups: <2 and ≥2 times/day according to “Times you brush your teeth in 1 day?”. Period since last dental visit was divided into three groups: <1, 1-2, and >2 years according to “When did you last visit a dentist?”. Fluoride drops/tablets were divided into three groups: no, yes, and unknown according to “Received Rx fluoride drops or tablets?”. Physical activity was divided into two groups: 7 and <7 days according to “Days physically active at least 60 min?” [30].

### Dental caries assessment

Dental caries experience was measured via NAHNES dental examiners using visual and tactile criteria to assess the status of each tooth for subjects aged ≥2 years old. Dental examiners were thoroughly trained in the NHANES examination protocol and exhibited high levels of inter-examiner reliability. Dental caries was the number of decayed or filled primary tooth surfaces (dfs) [32, 33]. The dfs was used as a cumulative measurement by summing the number of decayed (D), and filled (F) in 28 teeth. Primary tooth with a restored surface condition (A). Permanent tooth with a restored surface condition (F). Primary tooth with a dental carious surface condition (K). Permanent tooth with a dental carious surface condition (Z). Dental caries prevalence, the proportion of the children and adolescents with the dfs ≥1, was set as a dichotomous dependent variable.

### Statistical analysis

The weighted processing was carried out by SDMVPSU, SDMVSTRA and WTDR2D. The masked variance unit pseudo-stratum was SDMVSTRA, and the masked variance unit pseudo-primary sampling units (PSUs) was SDMVPSU. A set of adjusted weights, WTDR2D, is to be used when an analysis uses the smaller sample with completed Day 1 and Day 2 dietary data. Continuous variables were represented by mean ± standard error [Mean(±SE)], and intergroup comparison adopted Student’s *t* test. Categorical variables were described by the numbers and percentage [n (%)], and comparison between groups was performed by the Chi-square test or Kruskal-Wallis test.

Multiple imputation by chained equations (MICE) was used to missing data imputation. Sensitivity analysis was performed before and after missing data imputation (Table S1). The weighted univariate and multivariate logistic regression models were used to evaluate the association between total flavonoids, the subclasses intake and dental caries in children and adolescents, with odds ratios (ORs) with 95% confidence intervals (CIs). Covariates affecting dental caries including race, smoking, PIR, diabetes, antipsychotics, antidepressants, cotinine and neutrophils number were screened by univariate logistic regression model (Table S2). The associations in various subgroups of age, and overweight/obesity were further assessed. The association of anthocyanidins subclasses and catechins subclasses with dental caries in children and adolescents was further explored by stratified by age and individuals with overweight/obesity.

Data processing and statistical analyses were performed using Python 3.9.12 (Python Software Foundation, Delaware, USA) and SAS 9.4 (SAS Institute Inc., Cary, NC, USA). The *P*-value < 0.05 was regarded as statistically significant.

## Results

### Characteristics of the study population

**Fig. 1:**
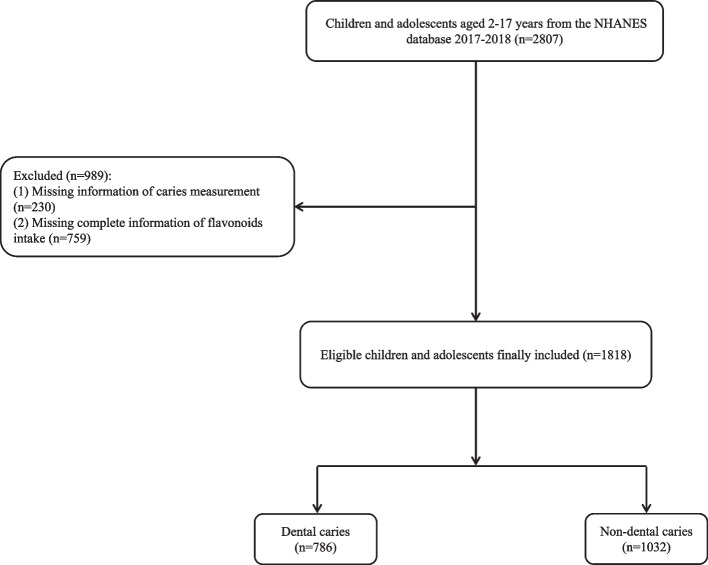
The screening flowchart of children and adolescents aged 2-17 years

The screening process was shown in Fig. 1. Totally 2,807 children and adolescents aged 2-17 years were screened. Among them, 230 participants with missing information of caries measurement, and 759 participants with missing complete information of flavonoids intake were excluded. Then 1,818 eligible children and adolescents were included, of which 786 (43.2 %) had dental caries. Table 1 shows the characteristics of the study population. The mean age of all participants was 9.64 (0.20) years old. There were significant differences between the two groups regarding age, race, PIR, household reference person education level, BMI, overweight/obesity, cotinine, total sugar, period since last dental visit, anthocyanidins, and flavan-3-ols (all* P* <0.05).

**Table 1:** The characteristics of study children and adolescents

Variables	Total (*n*=1818)	Non-dental caries (*n*=1032)	Dental caries (*n*=786)	Statistics	*P*
Age, years, Mean (S.E)	9.64 (0.20)	8.86 (0.23)	10.72 (0.28)	t=-5.74	<0.001
Age, years, Mean (S.E)				χ^2^=20.04	<0.001
<6	457 (23.91)	346 (30.74)	111 (14.40)		
≥6	1361 (76.09)	686 (69.26)	675 (85.60)		
Gender, n (%)				χ^2^=1.68	0.196
Male	894 (52.23)	494 (50.33)	400 (54.87)		
Female	924 (47.77)	538 (49.67)	386 (45.13)		
Race, n (%)				χ^2^=21.28	<0.001
Non-Hispanic White	592 (49.31)	369 (54.19)	223 (42.53)		
Non-Hispanic Black	438 (12.32)	263 (13.29)	175 (10.98)		
Others	788 (38.36)	400 (32.52)	388 (46.49)		
PIR, n (%)				χ^2^=4.60	0.032
<1.0	482 (22.85)	247 (20.26)	235 (26.45)		
≥1.0	1336 (77.15)	785 (79.74)	551 (73.55)		
Household reference person education level, n (%)				χ^2^=17.12	<0.001
Below high school	326 (15.21)	142 (10.52)	184 (21.73)		
High school and above	1492 (84.79)	890 (89.48)	602 (78.27)		
BMI, kg/m^2^, Mean (S.E)	20.19 (0.20)	19.59 (0.19)	21.02 (0.33)	t=-4.02	0.001
Overweight/obesity, n (%)				χ^2^=7.74	0.005
No	1122 (61.64)	662 (65.21)	460 (56.67)		
Yes	696 (38.36)	370 (34.79)	326 (43.33)		
Birth weight, pounds, n (%)				χ^2^=0.19	0.911
<5.5	271 (14.05)	161 (14.47)	110 (13.46)		
5.5-8.9	1415 (77.31)	795 (77.09)	620 (77.61)		
≥9	132 (8.64)	76 (8.43)	56 (8.93)		
Smoking during pregnancy, n (%)				χ^2^=0.85	0.355
No	1548 (85.01)	889 (86.24)	659 (83.30)		
Yes	270 (14.99)	143 (13.76)	127 (16.70)		
Serum cotinine, ng/mL, n (%)				χ^2^=13.21	0.001
≤0.05	784 (48.15)	435 (47.23)	349 (49.43)		
>0.05	612 (28.71)	297 (25.03)	315 (33.84)		
Unknown	422 (23.14)	300 (27.74)	122 (16.73)		
Total energy, kcal, Mean (S.E)	1802.92 (14.25)	1787.19 (20.48)	1824.80 (23.41)	t=-1.14	0.273
Total sugar, gm, Mean (S.E)	103.88 (1.32)	101.03 (1.62)	107.85 (2.40)	t=-2.33	0.034
Frequency of tooth brushing, times/day, n (%)				χ^2^=0.08	0.780
<2	602 (32.54)	337 (32.10)	265 (33.14)		
≥2	1216 (67.46)	695 (67.90)	521 (66.86)		
Amount of toothpaste use, n (%)				χ^2^=1.58	0.209
<Half load	620 (36.72)	373 (38.25)	247 (34.59)		
≥ Half load	1198 (63.28)	659 (61.75)	539 (65.41)		
Period since last dental visit, year, n (%)				χ^2^=38.30	<0.001
<1	1453 (81.04)	782 (77.18)	671 (86.43)		
1-2	144 (7.29)	72 (5.81)	72 (9.34)		
>2	221 (11.67)	178 (17.01)	43 (4.23)		
Fluoride drops/tablets, n (%)				χ^2^=0.27	0.874
No	1271 (67.36)	707 (66.57)	564 (68.46)		
Yes	159 (12.39)	86 (12.81)	73 (11.81)		
Unknown	388 (20.25)	239 (20.62)	149 (19.73)		
Physical activity, day, n (%)				χ^2^=11.45	<0.001
7	718 (39.35)	446 (43.65)	272 (33.37)		
<7	1100 (60.65)	586 (56.35)	514 (66.63)		
Total flavonoid, mg, Mean (S.E)	76.63 (6.24)	74.67 (7.31)	79.36 (7.95)	t=-0.54	0.597
Total flavonoid, mg, n (%)				χ^2^=0.64	0.726
≤24.60	624 (33.29)	344 (32.75)	280 (34.04)		
24.60-61.20	614 (33.40)	364 (34.50)	250 (31.86)		
>61.20	580 (33.31)	324 (32.74)	256 (34.10)		
Isoflavones, mg, Mean (S.E)	1.23 (0.45)	1.57 (0.72)	0.75 (0.17)	t=1.22	0.241
Isoflavones, mg, n (%)				χ^2^=0.74	0.692
0	767 (42.05)	424 (42.64)	343 (41.23)		
0-0.04	514 (28.69)	308 (29.36)	206 (27.76)		
>0.04	537 (29.26)	300 (28.00)	237 (31.01)		
Anthocyanidins, mg, Mean (S.E)	10.62 (1.70)	12.33 (2.58)	8.23 (1.28)	t=1.55	0.141
Anthocyanidins, mg, n (%)				χ^2^=9.24	0.010
≤0.42	623 (33.26)	327 (29.88)	296 (37.96)		
0.42-4.78	623 (33.41)	349 (33.43)	274 (33.38)		
>4.78	572 (33.33)	356 (36.69)	216 (28.66)		
Flavanones, mg, Mean (S.E)	13.23 (1.87)	11.95 (2.32)	15.02 (2.75)	t=-0.91	0.377
Flavanones, mg, n (%)				χ^2^=1.58	0.453
0	521 (27.93)	285 (28.23)	236 (27.51)		
0-3.16	678 (35.77)	403 (37.26)	275 (33.69)		
>3.16	619 (36.31)	344 (34.51)	275 (38.80)		
Flavonols, mg, Mean (S.E)	8.38 (0.38)	8.07 (0.37)	8.80 (0.56)	t=-1.43	0.174
Flavonols, mg, n (%)				χ^2^=4.79	0.091
≤4.27	613 (33.21)	358 (34.10)	255 (31.96)		
4.27-8.54	603 (33.48)	339 (35.67)	264 (30.44)		
>8.54	602 (33.31)	335 (30.23)	267 (37.60)		
Flavones, mg, Mean (S.E)	0.44 (0.03)	0.45 (0.03)	0.43 (0.05)	t=0.37	0.715
Flavones, mg, n (%)				χ^2^=2.10	0.350
≤0.10	607 (32.44)	334 (33.46)	273 (31.01)		
0.10-0.40	619 (34.21)	346 (32.24)	273 (36.96)		
>0.40	592 (33.35)	352 (34.30)	240 (32.03)		
Flavan-3-ols, mg, Mean (S.E)	42.73 (4.67)	40.30 (6.06)	46.12 (6.13)	t=-0.74	0.473
Flavan-3-ols, mg, n (%)				χ^2^=8.33	0.016
≤7.25	617 (33.25)	330 (30.26)	287 (37.40)		
7.25-19.52	589 (33.41)	357 (37.47)	232 (27.76)		
>19.52	612 (33.34)	345 (32.26)	267 (34.84)		
Subclass of flavan-3ols: catechins, mg, Mean (S.E)	19.77 (1.25)	19.98 (1.46)	19.47 (2.04)	t=0.21	0.834
Catechins, mg, n (%)				χ^2^=5.40	0.067
≤6.50	605 (33.21)	323 (29.64)	282 (38.18)		
6.50-16.85	580 (33.45)	343 (35.79)	237 (30.19)		
>16.85	633 (33.34)	366 (34.57)	267 (31.62)		
Subclass of flavan-3ols: theaflavins+ thearubigins, mg, Mean (S.E)	19.24 (3.07)	16.20 (3.42)	23.48 (4.32)	t=-1.57	0.136
Theaflavins+ thearubigins, mg, n (%)				χ^2^=1.55	0.460
0	1492 (82.04)	855 (82.99)	637 (80.73)		
0-58.95	146 (8.74)	83 (8.76)	63 (8.72)		
>58.95	180 (9.21)	94 (8.25)	86 (10.55)		

*t* t-test, *χ*^*2*^ Chi-square test, - Fisher exact, *S.E* Standard error, *PIR* Poverty income ratio, *BMI* Body mass index

### Associations of flavonoids intake with dental caries in children and adolescents

Table 2 shows the relationship between flavonoids intake and dental caries in children and adolescents. Compared with low intake of anthocyanidins (≤0.42 mg) and catechins (≤6.50 mg), intake of anthocyanidins (>4.78 mg) (OR=0.69, 95%CI: 0.52-0.92), and catechins (>16.85 mg) (OR=0.64, 95%CI: 0.44-0.92), were associated with lower odds of dental caries in children and adolescents, after adjustments for age, race, household education level, overweight/obesity, cotinine, total sugar, period since last dental visit, physical activity.

**Table 2:** Association of total flavonoids and the subclasses intakes with dental caries in children and adolescents

Variables	Crude model		Adjusted model*	
	OR (95% CI*)*	*P*	OR (95% CI)	*P*
Total flavonoid				
≤24.60 mg	Ref		Ref	
24.60 mg-61.20 mg	0.89 (0.57-1.39)	0.580	0.93 (0.57-1.52)	0.771
>61.20 mg	1.00 (0.68-1.47)	0.990	1.02 (0.67-1.56)	0.914
Isoflavones				
=0 mg	Ref		Ref	
0 mg-0.04 mg	0.98 (0.66-1.46)	0.906	1.01 (0.68-1.51)	0.949
>0.04 mg	1.15 (0.78-1.68)	0.462	1.07 (0.73-1.56)	0.717
Anthocyanidins				
≤0.42 mg	Ref		Ref	
0.42 mg-4.78 mg	0.79 (0.54-1.14)	0.187	0.78 (0.52-1.16)	0.201
>4.78 mg	0.61 (0.44-0.85)	0.007	0.69 (0.52-0.92)	0.014
Flavanones				
=0 mg	Ref		Ref	
0 mg-3.16 mg	0.93 (0.63-1.37)	0.688	0.92 (0.60-1.41)	0.691
>3.16 mg	1.15 (0.81-1.65)	0.409	1.07 (0.73-1.57)	0.697
Flavonols				
≤4.27 mg	Ref		Ref	
4.27 mg-8.54 mg	0.91 (0.59-1.41)	0.655	0.95 (0.63-1.43)	0.781
>8.54 mg	1.33 (0.98-1.80)	0.065	1.23 (0.86-1.75)	0.230
Flavones				
≤0.10 mg	Ref		Ref	
0.10 mg-0.40 mg	1.24 (0.85-1.80)	0.244	1.19 (0.81-1.74)	0.356
>0.40 mg	1.01 (0.71-1.44)	0.966	0.94 (0.62-1.41)	0.736
Flavan-3-ols				
≤7.25 mg	Ref		Ref	
7.25 mg-19.52 mg	0.60 (0.40-0.90)	0.017	0.60 (0.39-0.92)	0.021
>19.52 mg	0.87 (0.58-1.31)	0.488	0.85 (0.58-1.24)	0.372
Flavan-3ols subclass: catechins				
≤6.50 mg	Ref		Ref	
6.50 mg-16.85 mg	0.65 (0.42-1.01)	0.055	0.69 (0.45-1.06)	0.083
>16.8 mg	0.71 (0.47-1.07)	0.095	0.64 (0.44-0.92)	0.019
Flavan-3ols subclass: theaflavins+ thearubigins				
=0 mg	Ref		Ref	
0-58.95 mg	1.02 (0.61-1.70)	0.925	0.90 (0.54-1.49)	0.655
>58.95 mg	1.31 (0.84-2.06)	0.213	1.26 (0.81-1.96)	0.288

*Ref* Reference, *OR* Odd ratio, *CI* Confidence interval

^*^: adjusted for age, race, household education level, overweight/obesity, serum cotinine, total sugar, period since last dental visit, and physical activity

### Associations of anthocyanidins and catechins with dental caries in children and adolescents with different subgroups of age, and overweight/obesity

As summarized in Table 3, further analyses were conducted to explore the relationship between anthocyanidins and catechins and the risk of dental caries in age and overweight/obesity subgroups. The results showed that anthocyanidins (>4.78 mg) (OR=0.62, 95%CI: 0.43-0.90) and catechins (>16.85 mg) (OR=0.62, 95%CI: 0.40-0.96) were associated with lower odds of dental caries in children and adolescents aged ≥6 years. In addition, anthocyanidins (0.42 mg-4.78 mg, OR=0.53, 95%CI: 0.30-0.93; >4.78 mg, OR=0.58, 95%CI: 0.37-0.90), and catechins (6.50 mg-16.85 mg and >16.85 mg) (6.50 mg-16.85 mg, OR=0.45, 95%CI: 0.26-0.76; >16.85 mg OR=0.51, 95%CI: 0.31-0.84) were associated with lower odds of dental caries in children and adolescents without overweight/obesity.

**Table 3:** Association of anthocyanidins and catechins with dental caries in children and adolescents stratified by age, and overweight/obesity

Variables	Adjusted model			
	OR (95% CI)	*P*	OR (95% CI)	*P*
**Age**	**<6 years old**		**≥6 years old**	
Anthocyanidins				
≤0.42 mg	Ref		Ref	
0.42 mg-4.78 mg	0.77 (0.18-3.22)	0.698	0.82 (0.53-1.28)	0.354
>4.78 mg	1.29 (0.30-5.52)	0.714	0.62 (0.43-0.90)	0.014
Catechins				
≤6.50 mg	Ref		Ref	
6.50 mg-16.85 mg	0.42 (0.17-1.04)	0.059	0.76 (0.48-1.22)	0.241
>16.85 mg	0.68 (0.25-1.85)	0.422	0.62 (0.40-0.96)	0.033
**Overweight/obesity**	**No**		**Yes**	
Anthocyanidins				
≤0.42 mg	Ref		Ref	
0.42 mg-4.78 mg	0.53 (0.30-0.93)	0.030	1.36 (0.78-2.38)	0.250
>4.78 mg	0.58 (0.37-0.90)	0.019	0.90 (0.60-1.34)	0.566
Catechins				
≤6.50 mg	Ref		Ref	
6.50 mg-16.85 mg	0.45 (0.26-0.76)	0.006	1.25 (0.71-2.20)	0.414
>16.85 mg	0.51 (0.31-0.84)	0.012	0.88 (0.47-1.65)	0.677

*Ref* Reference, *OR* Odd ratio, *CI* Confidence interval

### Associations of anthocyanidins subclasses and catechins subclasses with dental caries in children and adolescents

**Fig. 2:**
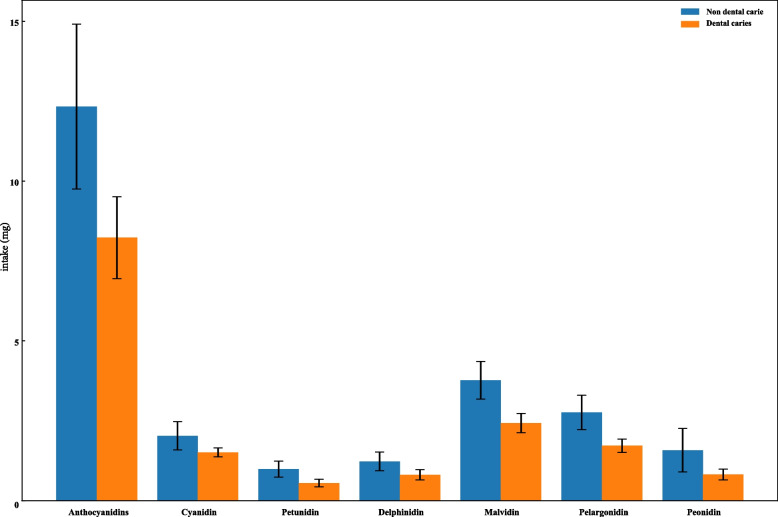
Total anthocyanidins and their subclasses intakes between dental caries and non- dental caries groups

**Fig. 3:**
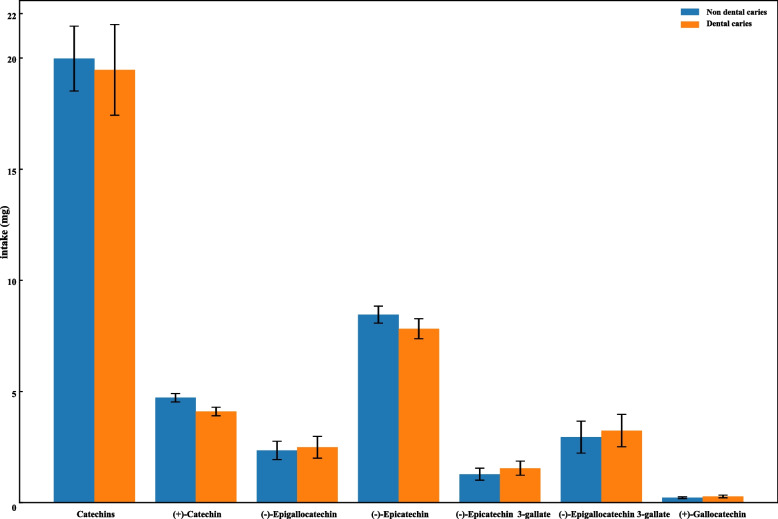
Total catechins and their subclasses intakes between dental caries and non- dental caries groups

To further examine the association of anthocyanidins subclasses and catechin subclasses with dental caries in children and adolescents, we first compared the differences in anthocyanidins subclasses and catechin subclasses between dental caries and non-dental caries groups. The results showed that there were significant differences between the dental caries group and non-dental caries group in cyanidin, petunidin, delphinidin, malvidin, pelargonidin, peonidin, (+)-catechin, (-)-epigallocatechin, (-)-epicatechin, (-)-epicatechin 3-gallate, (-)-epigallocatechin 3-gallate, (+)-gallocatechin (all *P* < 0.05) (Table 4, Figs. 2, and 3). Compared with low intake of anthocyanidins subclasses and catechins subclasses, intake of petunidin (0 mg<level≤0.27 mg and >0.27 mg) (0 mg-0.27 mg, OR=0.72, 95%CI: 0.58-0.89; >0.27 mg, OR=0.75, 95%CI: 0.56-0.98), malvidin (>1.06 mg, OR=0.70, 95%CI: 0.50-0.98), peonidin (>0.15 mg, OR=0.71, 95%CI: 0.51-0.97), (+)-catechin (>5.09 mg, OR=0.70, 95%CI: 0.52-0.94), (-)-epigallocatechin (0.04 mg-0.40 mg, OR=0.81, 95%CI: 0.67-0.98; >0.40 mg, OR=0.70, 95%CI: 0.55-0.90), and (-)-epicatechin (2.90 mg-9.61 mg, OR=0.62, 95%CI: 0.45-0.86; >9.61 mg, OR=0.71, 95%CI: 0.51-0.98), were associated with lower odds of dental caries in children and adolescents (Table 5).

**Table 4:** Comparisons of anthocyanidins subclasses and catechins subclasses between the two groups

Variables	Total (*n*=1818)	Non-dental caries (*n*=1032)	dental caries (*n*=786)	Statistics	*P*
Anthocyanidins					
Cyanidin				χ^2^=6.56	0.038
≤0.15 mg	639 (33.26)	342 (30.43)	297 (37.20)		
0.15 mg-1.44 mg	629 (32.99)	356 (32.39)	273 (33.82)		
>1.44 mg	550 (33.76)	334 (37.18)	216 (28.98)		
Petunidin				χ^2^=14.96	<0.001
=0 mg	919 (48.64)	483 (44.44)	436 (54.49)		
0 mg-0.27 mg	456 (25.35)	271 (27.26)	185 (22.69)		
>0.27 mg	443 (26.01)	278 (28.30)	165 (22.82)		
Delphinidin				χ^2^=7.82	0.020
=0 mg	887 (46.15)	469 (42.71)	418 (50.95)		
0 mg-0.45 mg	491 (27.12)	293 (28.36)	198 (25.39)		
>0.45 mg	440 (26.73)	270 (28.93)	170 (23.67)		
Malvidin				χ^2^=10.63	0.005
=0 mg	996 (52.46)	529 (48.13)	467 (58.49)		
0 mg-1.06 mg	404 (23.75)	231 (25.20)	173 (21.72)		
>1.06 mg	418 (23.79)	272 (26.67)	146 (19.78)		
Pelargonidin				χ^2^=6.09	0.048
=0 mg	906 (46.62)	494 (44.95)	412 (48.96)		
0 mg-0.32 mg	463 (26.59)	259 (25.59)	204 (27.98)		
>0.32 mg	449 (26.78)	279 (29.46)	170 (23.06)		
Peonidin				χ^2^=7.79	0.020
=0 mg	643 (34.59)	348 (31.44)	295 (38.97)		
0 mg-0.15 mg	594 (32.39)	325 (32.42)	269 (32.35)		
>0.15 mg	581 (33.02)	359 (36.14)	222 (28.68)		
Catechins					
(+)-Catechin				χ^2^=6.37	0.041
≤2.11 mg	592 (33.25)	320 (30.19)	272 (37.50)		
2.11 mg-5.09 mg	622 (33.43)	356 (34.56)	266 (31.86)		
>5.09 mg	604 (33.32)	356 (35.24)	248 (30.64)		
(-)-Epigallocatechin				χ^2^=9.61	0.008
≤0.04 mg	620 (32.88)	333 (30.06)	287 (36.79)		
0.04 mg-0.40 mg	619 (33.82)	357 (34.41)	262 (33.00)		
>0.40 mg	579 (33.30)	342 (35.53)	237 (30.20)		
(-)-Epicatechin, n (%)				χ^2^=8.73	0.013
≤2.90 mg	611 (33.29)	329 (30.09)	282 (37.75)		
2.90 mg-9.61 mg	605 (33.35)	358 (35.88)	247 (29.83)		
>9.61 mg	602 (33.36)	345 (34.03)	257 (32.42)		
(-)-Epicatechin 3-gallate, n (%)				χ^2^=6.69	0.035
=0 mg	700 (36.98)	374 (33.06)	326 (42.43)		
0 mg -0.07 mg	541 (30.90)	322 (33.62)	219 (27.12)		
>0.07 mg	577 (32.13)	336 (33.32)	241 (30.46)		
(-)-Epigallocatechin 3-gallate, n (%)				χ^2^=8.35	0.015
=0 mg	706 (36.00)	387 (33.05)	319 (40.10)		
0 mg-0.19 mg	576 (32.83)	345 (36.23)	231 (28.09)		
>0.19 mg	536 (31.17)	300 (30.72)	236 (31.81)		
(+)-Gallocatechin, n (%)				χ^2^=15.21	<0.001
=0 mg	1201 (66.69)	642 (62.29)	559 (72.83)		
0 mg-0.02 mg	301 (16.30)	198 (19.12)	103 (12.37)		
>0.02 mg	316 (17.01)	192 (18.59)	124 (14.80)		

*χ*^*2*^ Chi-square test, - Fisher exact

**Table 5:** Associations of anthocyanidins subclasses and catechins subclasses with dental caries in children and adolescents

Variables	Anthocyanidins		Variables	Catechins	
	OR (95%CI)	*P*		OR (95%CI)	*P*
Cyanidin			(+)-Catechin		
≤0.15 mg	Ref		≤2.11 mg	Ref	
0.15 mg-1.44 mg	0.88 (0.63-1.24)	0.451	2.11 mg-5.09 mg	0.78 (0.57-1.08)	0.128
>1.44 mg	0.72 (0.52-1.01)	0.054	>5.09 mg	0.70 (0.52-0.94)	0.021
Petunidin			(-)-Epigallocatechin		
=0 mg	Ref		≤0.04 mg	Ref	
0 mg-0.27 mg	0.72 (0.58-0.89)	0.005	0.04 mg-0.40 mg	0.81 (0.67-0.98)	0.036
>0.27 mg	0.75 (0.56-0.98)	0.039	>0.40 mg	0.70 (0.55-0.90)	0.008
Delphinidin			(-)-Epicatechin, n (%)		
=0 mg	Ref		≤2.90 mg	Ref	
0 mg-0.45 mg	0.80 (0.61-1.04)	0.086	2.90 mg-9.61 mg	0.62 (0.45-0.86)	0.007
>0.45 mg	0.77 (0.57-1.04)	0.082	>9.61 mg	0.71 (0.51-0.98)	0.041
Malvidin			(-)-Epicatechin 3-gallate, n (%)		
=0 mg	Ref		=0 mg	Ref	
0 mg-1.06 mg	0.76 (0.57-1.01)	0.055	0 mg -0.07 mg	0.74 (0.46-1.19)	0.193
>1.06 mg	0.70 (0.50-0.98)	0.041	>0.07 mg	0.80 (0.60-1.08)	0.131
Pelargonidin			(-)-Epigallocatechin 3-gallate, n (%)		
=0 mg	Ref		=0 mg	Ref	
0 mg-0.32 mg	1.09 (0.83-1.43)	0.496	0 mg-0.19 mg	0.72 (0.49-1.06)	0.093
>0.32 mg	0.83 (0.65-1.07)	0.144	>0.19 mg	0.90 (0.66-1.25)	0.511
Peonidin			(+)-Gallocatechin, n (%)		
=0 mg	Ref		=0 mg	Ref	
0 mg-0.15 mg	0.86 (0.63-1.15)	0.282	0 mg-0.02 mg	0.65 (0.42-1.02)	0.060
>0.15 mg	0.71 (0.51-0.97)	0.036	>0.02 mg	0.71 (0.50-1.02)	0.064

*Ref* Reference, *OR* Odd ratio, *CI* Confidence interval

## Discussion

This study aimed to investigate the association of flavonoids intake with dental caries in children and adolescents. Results showed that high intake of anthocyanidins and catechins were associated with lower odds of dental caries in children and adolescents. Similar results were discovered in patients aged ≥6 years, and without overweight/obesity. In addition, further studies found that high intake of cyanidin, petunidin, malvidin, peonidin, (+)-Catechin, (-)-Epigallocatechin, and (-)-epicatechin were associated with lower odds of dental caries in children and adolescents.

There are many risk factors for an individual to develop dental caries, of which poor dietary habits are important drivers [33, 34]. Sudies have confirmed that increasing dietary sources of flavonoids or taking supplements may promote dental remineralization and prevent the occurrence of dental caries [22, 23, 25]. Koyama et al. [26] showed that consumption of green tea with high concentrations of catechins, was associated with a lower odds of tooth loss. Grape seed extract (GSE) is an easily available plant-based supplement with a high concentration of proanthocyanidins. Delimont et al. [22] found that GSE inhibited the proliferation of bacterial biofilms on tooth surfaces and promoted dental remineralization. Most of the current studies have focused on adults as well as in vivo and in vitro experiments, and whether there is a benefit of flavonoid intake on dental caries in children and adolescents remains unclear. This study investigated the association of flavonoids intake with the risk of dental caries in children and adolescents. Our study found high intake of anthocyanidins and catechins were associated with lower odds of dental caries in children and adolescents. We further analyzed the association of the subclasses of anthocyanidins and catechin intake with the risk of dental caries in children and adolescents. Our study found that a diet loaded with high cyanidin, petunidin, malvidin, peonidin, (+)-catechin, (-)-epigallocatechin, and (-)-epicatechin, were associated with lower odds of dental caries in children and adolescents. Previous studies have demonstrated beneficial effects of anthocyanidins subclasses and catechin subclasses on dental caries [21, 25]. Vilela et al. found that green tea-derived epigallocatechin gallate (EGCG) reduced the number of S. mutans and might help prevent dental caries in children [21].

Our study showed that high intake of anthocyanidins and catechins were more beneficial for patients aged ≥6 years, reason for this may be that patients ≥6 years old form good lifestyle and diet habits [34]. Evidence suggests that poorer dental care habits, fewer dental care visits, excessive intake of fermentable carbohydrates, and increased intake sugar-rich foods contribute to an increased risk of dental caries in children [35–38]. Our study also indicated that the high intake of anthocyanidins and catechins were associated with lower odds of dental caries in children and adolescents without overweight/obesity. We speculate that the possible reason is that overweight/obesity patients may have a high consumption of refined carbohydrates, lipids, and low dietary fiber foods, whereas that these foods are positively associated with the incidence of dental caries [11, 12]. Due to the potential bias caused by the reduced sample size in these subgroups, larger specific populations are needed to validate these results in the future.

Several biological mechanisms may explain the association between anthocyanidins and catechins and dental caries. First, anthocyanidins and catechins decreased the growth and acid production of S. mutans by inhibiting phosphoenolpyruvate-dependent phosphotransferase system, (PEP-PTS) activity [22, 23]. Second, anthocyanidins and catechins have hydrophobic and hydrophilic properties, and can bind to a variety of compounds, especially minerals, proteins, and carbohydrates, and their binding can inhibit biofilm formation on the tooth surface [39, 40]. Third, anthocyanidins and catechins can inhibit bacteria-induced pH reduction by inhibiting glucose uptake into bacterial cells and thus bacterial metabolic activity, and maintain a neutral environment through their buffering capacity [22, 23].

This study possesses several strengths. Firstly, given that the incidence of dental caries in children and adolescents was increasing globally, our study was the first study to explore the association of flavonoid and its subclass intake with dental caries in such populations. Higher flavonoid intake, especially anthocyanidins and catechines, was found to be related to the risk of dental caries, which provides implications for the prevention of dental caries in children and adolescents. Secondly, in addition to common demographic information, in order to make our results more convincing, we adjusted as many covariates as possible that influence dental caries in children and adolescents in the multivariate logistic regression models. Finally, our study was based on a large population-based survey (NHANES) dataset, which employed a strictly random sampling process, ensuring that our results were representative of the entire population.

Several limitations require caution in interpreting our findings. First, this was cross-sectional, making it difficult to establish a causal association between flavonoids intake and the risk of dental caries. Second, due to patients with missing demographic and caries treatment-related information were excluded, this may have affected our results to some extent. Third, flavonoids intake was derived from dietary retrospective data, which may be affected by recall bias, and the results can be further validated by relevant biomarkers (such as plasma flavonoids levels) in the future. Fourth, due to the limitation of the database, detailed treatment information related to dental caries was not included in the analysis. Last but not least, our study focused on children and adolescents in the United States, and future large prospective cohort studies are needed to generalize our findings to other ethnic groups.

## Conclusion

High anthocyanidins and catechins intakes were associated with lower odds of dental caries in children and adolescents. It was indicated appropriate supplementation of anthocyanins and catechins may be beneficial in preventing dental caries in children and adolescents.

### Supplementary Information

Supplementary Material 1.

## Data Availability

The datasets generated and/or analyzed during the current study are available in the NHANES database, https://wwwn.cdc.gov/nchs/nhanes/.

## Abbreviations

NHANES: National Health and Nutrition Examination Survey
NCHS: National Center of Health Statistics
FNDDS: Food and Nutrient Database for Dietary Studies
PIR: Poverty income ratio
BMI: Body mass index
PSUs: Pseudo-primary sampling units
