# Supplementary material for: Association between flavonoids intake and dental caries in children and adolescents: a cross-sectional study from the NHANES database
Source: BMC Oral Health. 2024 Jul 26;24:848. doi: 10.1186/s12903-024-04567-1 (PMC11282690; doi:10.1186/s12903-024-04567-1)
Supplement: Supplementary file 1 — Supplementary Material 1. [file 12903_2024_4567_MOESM1_ESM.docx]

Table S1 Sensitivity analysis before and after data interpolation

| Variables | Before interpolation | After interpolation | Statistics | *P* |
| --- | --- | --- | --- | --- |
| Household reference person education level, n (%) |  |  | χ^2^=2.55 | 0.110 |
| <high school | 297 (14.65) | 326 (15.21) |  |  |
| ≥high school | 1436 (85.35) | 1492 (84.79) |  |  |
| PIR, n (%) |  |  | χ^2^=1.85 | 0.174 |
| <1.0 | 454 (23.27) | 482 (22.85) |  |  |
| ≥1.0 | 1211 (76.73) | 1336 (77.15) |  |  |
| WBC, 1000 cells/uL, Mean (S.E) | 7.33 (0.13) | 7.35 (0.12) | t=-0.50 | 0.623 |
| BMI, kg/m^2^, Mean (S.E) | 20.22 (0.21) | 20.19 (0.20) | t=1.65 | 0.119 |
| Birth weight, pounds, n (%) |  |  | χ^2^=0.72 | 0.696 |
| <5.5 | 223 (13.46) | 271 (14.05) |  |  |
| 5.5-9.0 | 1199 (77.93) | 1415 (77.31) |  |  |
| ≥9.0 | 112 (8.61) | 132 (8.64) |  |  |
| Smoking during pregnancy, n (%) |  |  | χ^2^=0.30 | 0.584 |
| No | 1341 (85.26) | 1548 (85.01) |  |  |
| Yes | 221 (14.74) | 270 (14.99) |  |  |
| Frequency of tooth brushing, n (%) |  |  | χ^2^=2.43 | 0.119 |
| <2 times/day | 558 (33.05) | 602 (32.54) |  |  |
| ≥2 times/day | 1114 (66.95) | 1216 (67.46) |  |  |
| Amount of toothpaste use, n (%) |  |  | χ^2^=0.63 | 0.428 |
| <Half load | 568 (37.09) | 620 (36.72) |  |  |
| ≥Half load | 1091 (62.91) | 1198 (63.28) |  |  |
| Period since last dental visit, n (%) |  |  | χ^2^=0.17 | 0.918 |
| <1 year | 1450 (81.06) | 1453 (81.04) |  |  |
| 1-2 years | 143 (7.29) | 144 (7.29) |  |  |
| >2 years | 220 (11.65) | 221 (11.67) |  |  |
| Physical activity, MET·min/week, n (%) |  |  | χ^2^=2.20 | 0.138 |
| =7 | 716 (39.49) | 718 (39.35) |  |  |
| <7 | 1090 (60.51) | 1100 (60.65) |  |  |

t: t-test; χ^2^: Chi-square test; PIR: poverty income ratio; WBC: white blood cell; BMI: body mass index; MET: metabolic equivalent task.

Table S2 Screening for potential covariates of dental caries

| Variables | OR (95%CI) | *P* |
| --- | --- | --- |
| Age |  |  |
| <6 | Ref |  |
| ≥6 | 2.64 (1.62-4.29) | <0.001 |
| Gender |  |  |
| Male | Ref |  |
| Female | 0.83 (0.62-1.13) | 0.215 |
| Race |  |  |
| Non-Hispanic White | Ref |  |
| Non-Hispanic Black | 1.05 (0.59-1.87) | 0.851 |
| Others | 1.82 (1.39-2.39) | <0.001 |
| PIR |  |  |
| <1.0 | Ref |  |
| ≥1.0 | 0.71 (0.49-1.02) | 0.060 |
| Household reference person education level |  |  |
| <high school | Ref |  |
| ≥high school | 0.42 (0.26-0.69) | 0.002 |
| Overweight/obesity |  |  |
| No | Ref |  |
| Yes | 1.43 (1.08-1.90) | 0.017 |
| Birth weight |  |  |
| <5.5 | Ref |  |
| 5.5-8.9 | 1.08 (0.65-1.81) | 0.747 |
| ≥9 | 1.14 (0.57-2.28) | 0.698 |
| Smoking during pregnancy |  |  |
| No | Ref |  |
| Yes | 1.26 (0.74-2.12) | 0.367 |
| Cotinine |  |  |
| ≤0.05 ng/mL | Ref |  |
| >0.05 ng/mL | 1.29 (0.91-1.83) | 0.135 |
| Unknown | 0.58 (0.36-0.91) | 0.022 |
| Total energy | 1.00 (1.00-1.00) | 0.272 |
| Total sugar | 1.01 (1.01-1.01) | 0.035 |
| Frequency of tooth brushing |  |  |
| <2 times/day | Ref |  |
| ≥2 times/day | 0.95 (0.67-1.37) | 0.784 |
| Amount of toothpaste use |  |  |
| <Half load | Ref |  |
| ≥Half load | 1.17 (0.89-1.54) | 0.232 |
| Period since last dental visit |  |  |
| <1 year | Ref |  |
| 1-2 years | 1.44 (0.74-2.77) | 0.261 |
| >2 years | 0.22 (0.14-0.37) | <0.001 |
| Fluoride drops/tablets |  |  |
| No | Ref |  |
| Yes | 0.90 (0.51-1.58) | 0.685 |
| Unknown | 0.93 (0.63-1.36) | 0.694 |
| Physical activity, MET·min/week, n (%) |  |  |
| =7 | Ref |  |
| <7 | 1.55 (1.16-2.07) | 0.006 |

Ref: reference, OR: odd ratio, CI: confidence interval; PIR: poverty income ratio; MET: metabolic equivalent task.
